# Intestinal helminthic infection and anemia among pregnant women attending ante-natal care (ANC) in East Wollega, Oromia, Ethiopia

**DOI:** 10.1186/s13104-017-2770-y

**Published:** 2017-09-05

**Authors:** Hylemariam Mihiretie Mengist, Olifan Zewdie, Adugna Belew

**Affiliations:** Department of Medical Laboratory Sciences, Faculty of Medical and Health Sciences, Wollega University, P.O.Box: 395, Nekemte, Ethiopia

**Keywords:** Intestinal helminths, Anemia, Pregnant women

## Abstract

**Background:**

Ethiopia is a developing country where intestinal helminthic infections are major public health problems. The burden of intestinal parasites, particularly the soil-transmitted helminths (STHs), is often very high in school children and pregnant women. Anemia, associated with STH, is a major factor in women’s health, especially during pregnancy; it is an important contributor to maternal mortality. The aim of this study was to determine the prevalence of intestinal helminthic infection and anemia among pregnant women attending ANC in East Wollega Zone, Ethiopia.

**Methods:**

A cross-sectional study was conducted in five health centers of East Wollega Zone of Oromia Region, Ethiopia between November 2015 and January 2016. The health centers were selected randomly and study participants were enrolled consecutively with proportions from all the health centers. Stool and blood specimens were processed using standard operating procedures in accordance with structured questionnaires. Logistic regression models were applied to assess the association between predictors and outcome variables. P values less than 0.05 were taken as significant levels. Results were presented in tables and figures.

**Results:**

A total of 372 pregnant women were enrolled in this study with a median age of 25 years (range 17–40 years). The total prevalence of intestinal helminths was 24.7% (92/372) with the predominance of Hookworm (15.1%) followed by *Ascaris lumbricoides* (6.5%). Illiteracy [AOR, 95% CI 2.21 (1.3, 4.8), P = 0.042], absence of latrine [AOR, 95% CI 4.62 (1.7, 8.3), P = 0.013] and regular consumption of raw and/or unwashed fruit [AOR, 95% CI 3.30 (1.2, 6.3), P = 0.011] were significant predictors of intestinal helminthic infection. The overall prevalence of anemia was 17.5% (65/372) where mild anemia accounts for 80% of the total anemia. Anemia was significantly associated with the first trimester of gestation [AOR, 95% CI 2.82 (1.3, 6.2), P = 0.009], previous malaria infection [AOR, 95% CI 2.32 (1.3, 5.3), P = 0.003], failing to take iron supplements regularly [AOR, 95% CI 1.82 (1.1, 4.8), P = 0.022] and infection with intestinal helminths specifically with Hook*worm* (P = 0.001) and *Ascaris lumbricoides* (P = 0.022).

**Conclusion:**

The prevalence of intestinal helminths and anemia was significantly high in this study. Different socio-demographic, lifestyle and obstetric factors were identified as significant contributors of intestinal helminthic infection and anemia among pregnant women. Therefore, public health measures and intensive antenatal care services are vital to promoting safe pregnancy.

## Introduction

Intestinal parasitic infection is a serious public health problem throughout the world, particularly in developing countries. *Amoebiasis, Ascariasis, Hookworm* infection and *Trichuriasis* are among the ten most common infections in the world [1].

Ethiopia is a developing country where intestinal parasitic infections (IPIs) are major public health problems. Previous studies carried out in Ethiopia revealed a high prevalence of IPIs [2, 3]. The burden of intestinal parasites, particularly the soil-transmitted helminths (STHs), is often very high in school children and pregnant women [4, 5].

Anemia is defined as a condition in which there is less than the normal hemoglobin (HB) level in the body, which decreases oxygen-carrying capacity of red blood cells to tissues. Anemia could be classified as mild, moderate and severe. It is more common in developing countries because of poor nutritional status and high prevalence parasitic infestation. Anemia is a major factor in women’s health, especially in developing countries. Severe anemia during pregnancy is an important contributor to maternal mortality [6]. For this particular study anemia was defined based on World Health Organization (WHO) anemia definition for pregnant women as anemia (hemoglobin level <11 g/dl), mild anemia (hemoglobin level between 10 and 10.9 g/dl), moderate anemia (hemoglobin level between 7.0 and 9.9 g/dl) and severe anemia (hemoglobin level <7.0 g/dl) [7].

Intestinal parasitic infections especially helminths increase anemia in pregnancy. The results of this are low pregnancy weight and intrauterine growth retardation, followed by low birth weight, with its associated greater risks of infection and higher prenatal mortality rates. An estimated 44 million pregnant women have hookworm infections which can cause chronic loss of blood from the intestines and predisposes the women to develop iron deficiency anemia [8].

Although there are many studies conducted in Ethiopia that have reported the magnitude of intestinal parasitic infections and anemia among pregnant women, there is a paucity of published data particularly on intestinal helminths and anemia in the study area. Furthermore, according to the federal ministry of health of Ethiopia, East Wollega zone is one of the highly Hookworm burdened districts in the country. The humid nature of the district which is suitable for intestinal helminths, the availability of street vended fruits and habit of wearing open shoe may increase the prevalence of intestinal helminths in East Wollega compared to other areas. The results of the study may help concerned stakeholders to take actions for the prevention of intestinal helminths and anemia in pregnant women. This study is, therefore, aimed to determine the prevalence of intestinal helminths and associated anemia among pregnant women in East Wollega.

## Specific objectives

The specific objectives of the research are;

- To determine the prevalence of intestinal helminthic infections among pregnant women attending ANC in East Wollega Zone, Ethiopia.
- To determine the prevalence of anemia among pregnant women attending ANC in East Wollega Zone, Ethiopia.
- To identify associated risk factors of intestinal helminthic infections attending ANC in East Wollega Zone, Ethiopia.
- To identify associated risk factors of anemia among pregnant women attending ANC in East Wollega Zone, Ethiopia.

## Hypothesis

The prevalence of anemia and intestinal helminths in the study area in not different from other areas.

## Methods

### Study setting and context

A cross-sectional study was conducted in randomly selected five health centers of East Wollega Zone of Oromia region, Ethiopia namely Jimma- Arjo health center, Arjo-Gudatu health center, Sire health center, Gute health center and Nekemte health center between November 2015 and January 2016. These study sites include both urban and rural areas.

### Sample size and sampling technique

Sample size was calculated using single population proportion formula considering 95% CI and P 0.41 [9] as follows;

$${\text{n}} = {\text{Z}}_{{{({\text{a}}/ 2)}}^{ 2}} {\text{P }}\left( { 1- {\text{P}}} \right)/{\text{d}}^{ 2} = 3 7 2$$

where n is sample size, d is the marginal error which is 0.05.

Finally, 372 pregnant women were enrolled consecutively from the five health centers where participants were allocated proportionally from all the health centers.

### Study population and data collection

Pregnant women taking anti-helminthic/anti-protozoan drugs within the past 2 weeks, very sick and unable to give information, with confirmed acute and/or chronic disease causing anemia were excluded. Site assessment and pre-test were done prior to data collection. Pregnant women were informed about the objective of the study before data collection and structured questionnaires were used to obtain data on socio-demographic, environmental related factors, obstetric characteristics, and dietary habits. The questionnaire was developed in English and participants were interviewed using local language; Afan Oromo. Medical Laboratory professionals who can speak the local language were trained on data collection for this particular study to attain standardization, maximize interviewer reliability and minimize bias. The data collectors were regularly supervised by the investigators for proper data collection. All reagents used were checked for their expiry date and prepared according to the manufacturer’s instructions.

### Specimen processing

A single stool specimen was collected from each participant. Freshly voided stool specimens were directly examined microscopically and preserved with 10% formalin for further analysis. Then preserved specimens were processed using formalin-ether concentration technique and examined microscopically for ova and larvae of helminths. Capillary blood was collected from a finger prick and hematocrit/hemoglobin was determined using the micro-hematocrit method. Anemia was defined based on WHO criteria as; anemia: hemoglobin level <11 g/dl for pregnant, mild anemia: hemoglobin level between 10 and 10.9 g/dl for pregnant women, moderate anemia: hemoglobin level between 7.0 nad 9.9 g/dl for pregnant women and severe anemia: hemoglobin level <7.0 g/dl for pregnant women.

#### Stool specimen collection and direct wet mount analysis

Stool specimen was obtained from all patients selected for the study. A direct saline and iodine wet mount of each sample were used to detect intestinal parasites microscopically. The wet mounts were examined under a light microscope at 100× and 400× magnifications [10].

#### Formol-ether concentration method

A portion of each preserved stool specimen was taken and processed following standard procedures to determine intestinal helminths. Briefly, 1 g of stool was placed in a clean conical centrifuge tube containing 7 ml 10% formol water by using applicator stick. The resulting suspension was filtered through a sieve into another conical tube. After adding 3–4 ml of diethyl ether to the formalin solution, the content was centrifuged at 3200 rpm for 1 min. The supernatant was discarded; smear was prepared from the sediment and observed under a light microscope with a magnification of 100× and 400× after air dried [10].

### Data quality assurance

All laboratory analyses were carried out using standard operating procedures.

#### Pre-analytical

An adequate stool and blood specimens were collected. Carefully labeled, dry, leak-proof, grease free transparent stool caps were used to collect stool. The specimens were kept free of contamination from water, soil, and urine. Specimens contaminated with water, urine and soil were rejected.

#### Analytical

Direct stool examination was performed within 30 min of collection and an appropriate amount of stool sample was used to make a good smear devoid of air bubbles. After checking the contrast using 10× objective, the ova and larva of helminths were examined using 40× objective. Each stool smear was examined for at least 10–15 min. hemoglobin level was determined based on standard procedures.

#### Post-analytical

All microscopic findings and hemoglobin level were encoded and reported appropriately.

### Statistical analysis

The data was cleaned, coded and double entered. EpiData version 3.1 was used for data entry. Then data was exported to SPSS version 20 (SPSS INC, Chicago, IL, USA) software for analysis. Missing data were managed by observing cross tabulation results and percentages. Logistic regression models and one-way ANOVA were used to determine the association between predictors and outcome variables. Binary logistic regression was used to see the crude association between dependent and explanatory factors and multinomial logistic regression was used to adjust for confounders. P values less than 0.05 were taken as statistically significant. Results were presented using tables and figures and conclusion and recommendations were derived accordingly.

## Results

### Socio-demographic characteristics of study participants

A total of 372 eligible pregnant women participated in this study. The median age of the participants was 25 years with a range of 17–40 years. The majority (78.8%) of participants were below 29 years of age while 236 (63.4%) of pregnant women were rural residents. Regarding education, 127 (34.1%) of the participants were illiterates (no formal education). Most of the study participants; 92.5 and 88.7% were married and earn an average monthly income below 1500 Ethiopian Birr, respectively while 173 (46.5%) were farmers (Table 1).

**Table 1:** Socio-demographic characteristics of pregnant women attending ANC in Five Health centers from November 2015 to January 2016, East Wollega, Ethiopia

Characteristics	Number	Percent
Age group		
≤29	293	78.8
>29	79	21.2
Residence		
Rural	236	63.4
Urban	136	36.6
Educational status		
Illiterate	127	34.1
Read and write	136	36.6
Primary school	48	12.9
High school and above	61	16.4
Marital status		
Single	7	1.9
Divorced	20	5.4
Widowed	1	0.3
Married	344	92.5
Average monthly income		
ETB < 1500	330	88.7
ETB ≥ 1500	42	11.3
Occupation		
Farmer	173	46.5
Housewife	145	39
Civil servant	15	4
Others	39	10.5

### Prevalence of intestinal helminths and associated risk factors

**Fig. 1:**
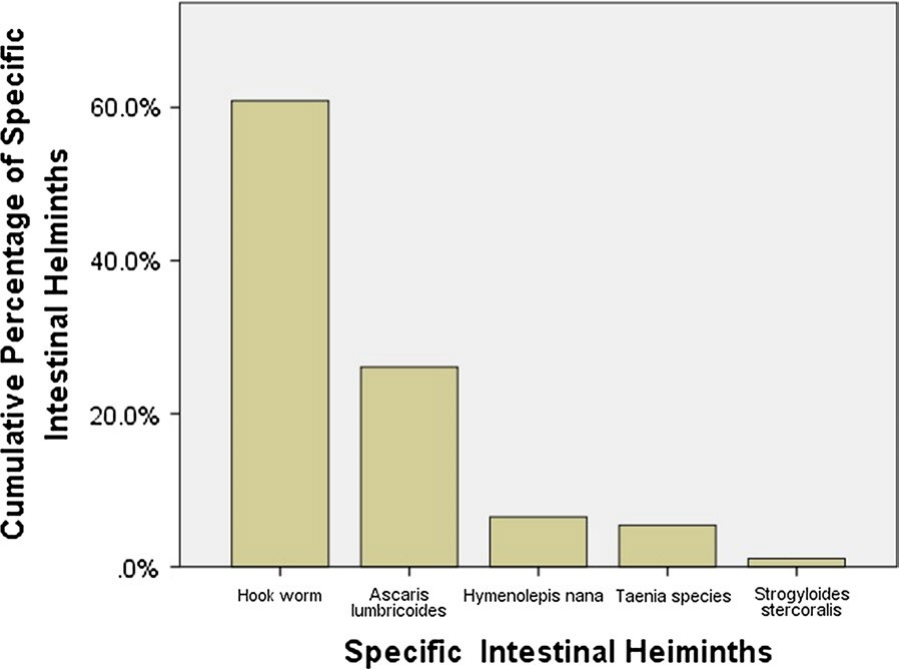
Distribution of species-specific intestinal helminths among pregnant women attending ANC in East Wollega, Oromia, Ethiopia from November 2015 to January 2016

The total prevalence of intestinal helminths in this study was 24.7% (92/372). Four (1.1%) of pregnant women were infected with two and more intestinal helminths. The predominant intestinal helminth was Hookworm which accounted for 15.1% (56/372) followed by *Ascaris lumbricoides* 6.5% (24/372), *Hymenolepis nana* 1.6% (6/372), *Taenia species* 1.3% (5/372) and *Strongyloides stercoralis* 0.3% (1/372) (Fig. 1).

Among the total study participants, one-fifth (75/372) of them lack functional latrine and 40% (30/75) of them were infected with intestinal helminths. Around 87.4% (325/372) of study participants had been washing their hands after latrine and a quarter of them (24.6%) had an intestinal helminthic infection. Open field defecation had been practiced by 39.5% (147/372) of respondents and 26.5% (39/147) of them harbored intestinal helminths. Around 49% (181/372) of pregnant women were using unprotected source of drinking water and 28% (51/181) of them were infected with intestinal helminths (Table 2).

**Table 2:** Assessment of risk factors associated with intestinal helminths among pregnant women attending ANC in East Wollega, Oromia, Ethiopia from November 2015 to January 2016

Variables	Helminthes		COR (95% CI)	P	AOR (95% CI)	P
	Present, N (%)	Absent, N (%)				
Age group						
≤29 years	67 (22.8)	226 (77.2)	1		1	
>29 years	25 (31.6)	54 (68.4)	0.64 (0.4, 1.1)	0.112	1.32 (0.7, 2.3)	0.453
Residence						
Rural	63 (36.4)	173 (63.6)	1.34 (0.8, 2.2)	0.436		
Urban	29 (21.3)	107 (78.7)	1			
Educational status						
Illiterate	39 (30.7)	88 (69.3)	1.61 (1.3, 6.8)	0.014*	2.21 (1.3, 4.8)	0.042*
Literate	53 (21.6)	192 (78.4)	1		1	
Presence of latrine						
Absent	30 (40)	45 (60)	2.53 (1.5, 4.3)	0.001*	4.62 (1.7, 8.3)	0.013*
Present	62 (21)	235 (79)	1		1	
Hand washing after latrine						
No	12 (25.5)	35 (74.5)	1.00 (0.5, 2.1)	0.894		
Yes	80 (24.6)	245 (75.4)	1			
Open field defecation						
Yes	39 (26.5)	108 (73.5)	1.23 (0.7, 1.9)	0.522		
No	53 (23.5)	172 (76.5)	1			
Hand washing before meal						
No	9 (24.3)	28 (75.7)	0.98 (0.4, 2.1)	0.924		
Yes	83 (24.8)	252 (75.2)	1			
Source of drinking water						
Unprotected	51 (28.2)	130 (71.8)	1.43 (0.9, 2.3)	0.142	1.04 (0.6, 1.8)	0.872
Protected	41 (21.5)	150 (78.5)	1		1	
Wearing closed shoe regularly						
No	55 (23.6)	178 (76.4)	0.85 (0.3, 1.4)	0.526		
Yes	37 (26.6)	102 (73.4)	1			
Eating raw meat						
Yes	72 (26.2)	203 (73.8)	1.37 (0.8, 2.4)	0.285		
No	20 (20.6)	77 (79.4)	1			
Eating raw fruit						
Yes	74 (24.3)	231 (75.7)	8.8 (1.5, 13.6)	0.033	3.30 (1.2, 6.3)	0.011*
No	18 (26.9)	49 (73.1)	1		1	
Close contact with pet animals						
Yes	56 (30)	131 (70)	1.77 (1.1, 2.8)	0.022*	0.73 (0.4, 1.2)	0.223
No	36 (19.5)	149 (80.5)	1		1	
Eating soil						
Yes	19 (29.7)	45 (70.3)	1			
No	73 (23.7)	235 (76.3)	1.36 (0.8, 2.5)	0.343		

*COR* crude odds ratio, *AOR* adjusted odds ratio

*Statistically significant P values

Comparatively intestinal helminths were more prevalent in rural residents than urban residents (36.4% versus 21.3%); although, residence was not a significant risk factor (P = 0.436). The prevalence of intestinal helminths was significantly higher among illiterates where 30.7% (39/127) of them were infected [AOR, 95% CI 2.21 (1.3, 4.8), P = 0.042] (Table 2).

Absence of latrine and eating raw/unwashed fruit were significant predictors of intestinal helminthic infection where pregnant women lacking latrine and consuming raw/unwashed fruit regularly were 4.6 and 3.3 times more often infected with intestinal helminths than their counterparts ([AOR, 95% CI 4.62 (1.7, 8.3), P = 0.013], [AOR, 95% CI 3.30 (1.2, 6.3), P = 0.011]), respectively. Open field defecation, hand washing habit, eating raw/undercooked meat, the habit of wearing closed shoe and eating soil was not significantly associated with the prevalence of intestinal helminths in binary logistic regression (P > 0.05) (Table 2).

### Assessment of anemia and risk factors

The overall prevalence of anemia in this study was 17.5% (65/372) with a mean hemoglobin level of 12.2 ± 1.5 g/dl. The predominant type of anemia was mild anemia which accounted for 80% (52/65) of the total anemia followed by moderate and severe anemia.

**Fig. 2:**
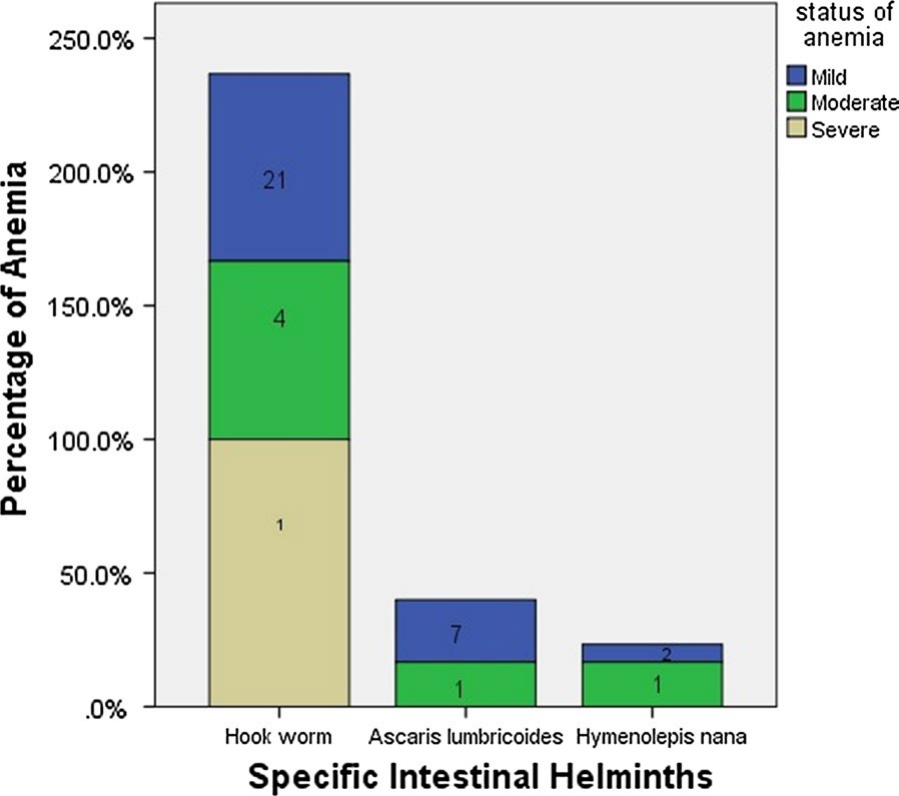
Distribution of status of anemia by species-specific intestinal helminths among pregnant women attending ANC in East Wollega, Oromia, Ethiopia from November 2015 to January 2016

Figure 2 shows that anemia was more prevalent in pregnant women infected with Hookworms and *Ascaris lumbricoides*. Severe anemia was not reported from pregnant women not infected with intestinal helminths.

The prevalence of anemia in rural and urban residents was almost similar (17.4% versus 17.6%). Anemia was higher in illiterates (19%) and those aged above 29 years (21.5%). Fifty-one (13.7%) of pregnant women were in their first trimester of gestational age and 35.3% of them were anemic. Taking iron supplements regularly was reported by 51.6% (192/372) of pregnant women (Table 3).

**Table 3:** Assessment of risk factors associated with anemia among pregnant women attending ANC in East Wollega, Oromia, Ethiopia from November 2015 to January 2016

Variables	Anemia		COR (95% CI)	P	AOR (95% CI)	P
	Present, N (%)	Absent, N (%)				
Residence						
Rural	41 (17.4%)	195 (82.6%)	0.98 (0.6, 1.7)	0.952		
Urban	24 (17.6%)	112 (82.4%)	1			
Age group						
≤29 years	48 (16.4%)	245 (83.6%)	1			
>29 years	17 (21.5%)	62 (78.5%)	0.73 (0.4, 1.3)	0.334		
Educational status						
Illiterate	24 (19%)	103 (81%)	2.61 (0.9, 7.2)	0.071		
Literate	41 (16.7%)	204 (83.3%)	1			
Food diversity						
No	13 (25.5)	38 (74.5)	1.73 (0.8, 3.5)	0.043	0.63 (0.3, 1.4)	0.336
Yes	52 (16.5)	269 (83.5)	1		1	
Coffee after meal						
Yes	52 (17)	254 (83)	0.87 (0.4, 1.7)	0.887		
No	13 (20.3)	51 (79.7)	1			
Home delivery						
Yes	15 (15.2)	84 (84.8)	0.74 (0.3, 1.3)	0.326		
No	42 (21.4)	154 (78.6)	1			
Gestational age (Trimester)						
First	18 (35.3)	33 (64.7)	0.32 (0.1, 0.6)	0.001*	2.82 (1.4, 6.2)	0.009*
Second	18 (15.8)	96 (84.2)	0.87 (0.5, 1.6)	0.667		
Third	29 (14)	178 (86)	1		1	
Following regular ANC						
No	7 (16.7)	35 (83.3)	0.93 (0.4, 2.2)	0.886		
Yes	58 (17.6)	272 (82.4)	1			
Birth interval						
<2 years	13 (21.7)	47 (78.3)	1.22 (0.6, 2.4)	0.776		
>2 years	44 (19)	188 (81)	1			
Presence of current blood loss						
Yes	20 (29.4)	48 (70.6)	2.42 (1.3, 4.4)	0.007*	0.73 (0.4, 1.5)	0.351
No	45 (14.8)	259 (85.2)	1		1	
Previous Malaria infection						
Yes	25 (35.2)	46 (64.8)	3.53 (1.9, 6.4)	<0.001	2.32 (1.3, 5.3)	0.003*
No	40 (13.3)	261 (86.7)	1		1	
Using anti-malaria bed net						
No	26 (17.3)	124 (82.7)	0.94 (0.6, 1.7)	0.952		
Yes	39 (17.6)	183 (82.4)	1			
Taking iron supplements regularly						
No	24 (13.3)	156 (86.7)	0.64 (0.3, 0.9)	0.04*	1.82 (1.1, 4.8)	0.022*
Yes	41 (21.4)	151 (78.6)	1		1	
Intestinal helminths						
Present	37 (40.2)	55 (59.8)	6.11 (3.2, 9.7)	<0.001	3.12 (1.5, 7.3)	0.023*
Absent	28 (10)	252 (90)	1		1	
*Hook worm*						
Present	26 (44.8)	32 (55.2)	5.70 (3.1, 9.6)	<0.001	3.53 (1.6, 6.7)	0.001*
Absent	39 (12.4)	275 (87.6)	1		1	
*Ascaris lumbricoides*						
Present	9 (34.6)	17 (65.4)	2.70 (1.2, 6.5)	0.031*	1.82 (1.1, 3.8)	0.022*
Absent	56 (16.2)	290 (83.8)	1		1	

*COR* crude odds ratio, *AOR* adjusted odds ratio

*Statistically significant P values

In binary logistic regression; taking coffee immediately after a meal, presence of blood loss in current pregnancy, malaria infection within the last 1 year and failing to take iron supplements regularly were significantly associated with anemia (P < 0.05). After adjusting confounders, previous malaria infection within the last 1 year [AOR, 95% CI 2.32 (1.3, 5.3), P = 0.003], first trimester of gestational age [AOR, 95% CI 2.82 (1.4, 6.2), P = 0.009] and failing to take iron supplements regularly [AOR, 95% CI 1.82 (1.1, 4.8), P = 0.022] were found to be significant causes of anemia. Anemia was more prevalent among pregnant women infected with Hookworm [AOR, 95% CI 3.53 (3.01, 9.6), P = 0.001] and *Ascaris lumbricoides* [AOR, 95% CI 1.82 (1.2, 6.5), P = 0.022] (P = 0.02) (Table 3). In one-way ANOVA analysis, there was a significant mean difference of hemoglobin between intestinal helminth-infected and non-infected pregnant women (F = 45, P = 0.001).

In summary, the odds of developing anemia were; 2.82, 3.12, 1.82 and 2.32 times more likely among pregnant women with their first trimester of gestation, infected with intestinal helminths, those taking iron supplements irregularly and infected with malaria within the last 1 year, respectively (Table 3).

## Discussion

Ethiopia is a developing country where IPIs are major public health problems. Previous studies carried out in Ethiopia revealed a high prevalence of IPIs [2, 3]. The burden of intestinal parasites, particularly the soil-transmitted helminths (STHs), is often very high in school children and pregnant women [4, 5]. Hookworm is reported to be highly prevalent in Illubabor, Kaffa and Wollega districts of Ethiopia.

The prevalence of intestinal helminths in pregnant women in this study (24.7%) is lower than data from South West Ethiopia (41%) [9] and Nigeria (48.3%) [11]. But it is higher than a study done in Kenya (13.8%) [12] and Ethiopia (13.7%) [13]. It is again lower than data from Bushlo Health Center, Ethiopia (58.3%) [14] and Hossana, Sothern Ethiopia 29.4% [15]. This difference could be due to geographic differences, lack of sensitive techniques to diagnose different intestinal helminths in the present study which may hinder the results, better awareness of pregnant women about ANC follow up, proper cooking of food or better personal hygiene in these days in the present study.

The predominant intestinal helminth in this study was Hookworm (15.1%) followed by *Ascaris lumbricoides* (6.5%), *Hymenolepis nana* (1.6%), *Taenia species* (1.3%) and *Strongyloides stercoralis* (0.3%). Higher prevalence of Hookworm in the present study is similar to other studies conducted in Ethiopia [9, 14, 15] which strengthen the reports stating high prevalence of Hookworm in Southern and Western districts of Ethiopia. Conversely, this predominance of Hookworm in this study is different from studies conducted in Nigeria [11] and Kenya [12] where authors reported as *Ascaris lumbricoides* was the predominant intestinal helminth. The possible difference could be due to the geographic difference, tropical nature of our study area which favors Hookworm transmission and difference in the habit of walking barefoot which is more practiced in Ethiopia.

Illiteracy, the absence of latrine and eating raw/unwashed fruit were independent predictors of intestinal helminthic infection in the present study. Except for educational status, this result is different from a study from Kenya [12] where authors reported age and hand washing habit were significant factors for intestinal helminthic infection. This difference might be due to a high level of illiteracy, lack of latrine facilities and increased consumption of street vended fruits in Ethiopia.

On the other hand, a research was done in Hosana, Southern Ethiopia [15] reported walking barefooted, unprotected source of water, rural residence and low monthly income (<1500 birr) had a positive association with intestinal helminths which is different from the present study. The possible explanation for this difference could be due to the comparable prevalence of intestinal helminths in both rural and urban residents and a higher percentage of pregnant women using protected water source in the current study.

Anemia in pregnancy should be strictly managed and prevented as recommended by world health organization (WHO) [16]. The total prevalence of anemia in this study was 17.5%. This figure is very low compared to previous studies in Ethiopia [13, 17], Ghana [18], Malawi (57.1%) [19], Peru (50%) [20], West Arsi (36.6%) [21] and Eastern Ethiopia (58.6%) [22]. But comparative with data from Northwest Ethiopia (21.6%) [23] and Addis Ababa (21.3%) [24]. This difference could be explained by comparative oldness of the previous data, large study area in the previous studies, unable to use sensitive techniques to determine hemoglobin level in the current study, difference in altitude and better awareness of pregnant women in including diversified food diets, using anti-malaria bed net and prevention of intestinal helminths in recent times.

The predominance of mild anemia (hemoglobin level 10–10.9 g/dl) in this study is in agreement with studies from different parts of Ethiopia [22, 24–26]. In this study, anemia was significantly associated with intestinal helminthic infection specifically with Hookworm and *Ascaris lumbricoides*, previous malaria infection and irregular use of iron supplements.

The prevalence of anemia in rural and urban residents was similar in this study which is different from studies in Jimma, Ethiopia [17] and Malawi [19]. This disagreement could be due to similar health care facility setup in both residences in this study since the data was collected in Woreda health centers. Similar to other studies from Ethiopia [13, 14, 23, 26] anemia was significantly associated with intestinal helminthic infection in this study.

The significant association of anemia with Hookworm infection in this study agrees with findings in different parts of Ethiopia [14, 17, 23, 25, 26]. This agreement can have the power to initiate all health care administrators from different regions of the country to take actions to prevent Hookworm infection and thus prevent complications of anemia during pregnancy. Gestational age was one of the significant predictors of anemia in the present study similar to different studies reported from Ethiopia [21, 22, 24]; although, first, second or third trimesters were significantly associated with anemia in the previous studies while only first trimester was a significant predictor of anemia in the present study.

The increased likely hood of being anemic in pregnant women with previous malaria infection in this study is similar to previous studies conducted in Ethiopia [23, 26]. Further anemia was significantly higher in pregnant women who failed to take iron supplements regularly in the present study which is in agreement with studies conducted in different districts of Ethiopia [13, 22, 23].

Data from different parts of Ethiopia again [14, 23–26] reported age group, rural residence, low average monthly income, chronic illness and birth interval were significant risk factors for anemia which is different with the present study. The inability of assessing chronic illnesses, altitude difference, the comparable prevalence of anemia in both rural and urban residents in both age groups and lack of accurate techniques to determine hemoglobin level in our study setting could be the possible justifications of the differences.

In this study, we have used formol-ether concentration technique for detection of intestinal helminths. We have not used floatation techniques and other sensitive methods which might result underestimation of the prevalence of intestinal helminths. Further, we have not used automated machines to measure hemoglobin level which may again render the prevalence of anemia in this study.

## Conclusion

The prevalence of intestinal helminths in the study area was significantly high. Five different species of intestinal helminths were identified with a predominance of soil-transmitted helminths (*Hookworm* and *Ascaris lumbricoides*). Illiteracy, the absence of latrine and consumption of raw and/or unwashed fruits were identified as independent risk factors that significantly increase intestinal helminths in pregnant women. The prevalence of anemia was high with a predominance of the mild type. Anemia was significantly high among pregnant women with the first trimester of gestation, infected with malaria in the previous 1 year, take iron supplements irregularly and infected with intestinal helminths specifically with *Hookworm* and *Ascaris lumbricoides*. Public health measures, regular ANC follow up with an intensive diagnosis of intestinal helminths and further longitudinal studies are recommended.

## Abbreviations

ANC: antenatal care
AOR: adjusted odds ratio
COR: crude odds ratio
IRB: Institutional review board
STHs: soil-transmitted helminths
WHO: World Health Organization
